# Acceptance of a New Food Enriched in β-Glucans among Adolescents: Effects of Food Technology Neophobia and Healthy Food Habits

**DOI:** 10.3390/foods8100433

**Published:** 2019-09-23

**Authors:** Cristina Proserpio, Ella Pagliarini, Monica Laureati, Beatrice Frigerio, Vera Lavelli

**Affiliations:** Department of Food, Environmental and Nutritional Sciences (DeFENS), University of Milan, 20133 Milan, Italy; ella.pagliarini@unimi.it (E.P.); monica.laureati@unimi.it (M.L.); beatrice.frigerio1@studenti.unimi.it (B.F.); vera.lavelli@unimi.it (V.L.)

**Keywords:** β-glucans, *Pleurotus*, functional food, liking, food neophobia, healthy behavior

## Abstract

The aim of the present study was to evaluate adolescents’ acceptability of a novel flat bread modified by substituting a part of the wheat flour content with a *Pleurotus ostreatus* powder rich in β-glucans, which can potentially provide health benefits. The effects of food technology neophobia and adolescents’ food habits on hedonic perception of the developed product was also investigated. Two hundred and two adolescents (age range: 13–18 years; girls: 49.5%; boys: 50.5%) evaluated their liking of two flat breads, one with mushroom powder added and one control sample with only wheat flour. Sample acceptance was studied in relation to age, gender, neophobic traits and healthy food habits. The results showed that, even if the sample with mushroom powder added was generally well accepted, there were different hedonic responses among adolescents according to their food technology neophobia level and healthy habits. In particular, adolescents with a low food technology neophobia level and healthy eating behavior mostly appreciated the sample with mushroom powder added, whereas subjects with neophobic and unhealthy eating behavior gave comparable hedonic scores to the two samples. Moreover, a negative correlation was found between food technology neophobia level and healthy food habits. In conclusion, it is possible to develop a β-glucan-enriched product appreciated by adolescents using a sustainable ingredient. The developed product may be used to achieve the daily recommended intake of β-glucans by adolescents.

## 1. Introduction

It is well known that a proper dietary intake and healthy lifestyle are associated with a lower risk of pathologies, such as obesity and type-2 diabetes, in both children and adolescents. The overweight condition, if not properly treated, continues into adulthood, resulting in other pathologies such as cardiovascular diseases [1]. Potential health benefits have been widely associated with the consumption of an appropriate amount of fiber [2]. However, results of previous studies highlighted that the total dietary fiber intake is rather low among European adolescents compared to the World Health Organization recommendations. Moreover, it has been reported that the significant reduction in fiber consumption observed in industrialized countries is related to a worrying increase in adolescent obesity [3].

Dietary fiber includes heterogeneous classes of compounds, such as oligosaccharides and resistant starch, that may be associated with lignin and other non-carbohydrate components (e.g., polyphenols, saponins, and resistant protein) [4]. Among these compounds, water-soluble fiber (WSF) purportedly has various positive health effects. Indeed, WSF is able to hold up bowel absorption, which can reduce cholesterol absorption due to the development of viscous solutions in the gastrointestinal tract [5]. Moreover, WSF fermentation can produce short-chain fatty acids, leading to longer lasting satiety, a decreased acute insulin response and a lower glycemic index of the consumed food [5,6].

Among the WSFs, β-(1→3)- and β-(1→6) β-glucans in mushrooms have raised attention due to their specific health properties [7,8]. In particular, the *Pleurotus ostreatus* mushroom shows a high total glucan content in the range of 14–25% d.w., with a percentage of β-glucans between 73% and 91.4% [9]. *P. ostreatus* strains with β-glucans up to 32.2% d.w. have also been identified [10].

Previous studies have investigated the effects of dietary supplementation with powder from *Pleurotus* spp. in both healthy and unhealthy humans. Daily doses of 3–5 g of *P. ostreatus* powder have been found to decrease the fasting plasma glucose level in both healthy people and people affected by type-2 diabetes [11,12]. Additionally, purification of the β-glucan fraction has led to the isolation of insoluble pleuran. Pleuran has been proven to be an effective immunostimulant at a 100 mg/day dose in young athletes who have depressed immune systems due to excessive and exhausting physical loads [13,14]. Moreover, the immunomodulatory properties of pleuran have been found to be effective in children with respiratory diseases [15]. Hence, the effective amount of *P. ostreatus* in vivo supports its use as an ingredient in functional foods.

The importance of *P. ostreatus* is also associated with its ability to grow on low-cost substrates, including wood and waste of the agri-food system [16,17], which makes it a sustainable food. However, it is crucial to understand the response of consumers toward the use of this mushroom as a functional ingredient in new food formulations, since it could be considered as unfamiliar due its lower consumption in Europe compared to other more common species, such as *A. bisporus*. Indeed, 80% of *P. ostreatus* production occurs in Asia, while in the other continents the production and use of this mushroom are quite low [16].

Only a few studies have investigated the acceptance of consumers of *P. ostreatus* as a new food ingredient, and none of these studies were specifically aimed at evaluating the acceptability toward new food formulations by adolescents. Nowadays, adolescents are less influenced by the family environment in their food choices and preference; therefore, they play their own role in the food consumption process [18], representing a large and important market segment [19]. However, their food behavior is often discussed, but not completely understood. For this reason, it is interesting to gain insight into which factors influence their behavior and involvement with food and healthy lifestyles. Various findings have highlighted that adolescents are influenced by what their peers eat [20], leading to a higher risk of unhealthy behavior [21]. Literature results have shown that an efficient tool to measure healthy eating behavior in adolescents is the Adolescent Food Habits Checklist (AFHC), developed by Johnson and colleagues [22]. This tool allows the evaluation of food choice situations in which adolescents are likely to have a degree of personal control and it is mainly focused on the consumption of energy dense foods and fruit and vegetable intake. Another factor that could influence consumers’ behavior is the fear of new food technologies (Food Neophobia Technology) for the development of novel and functional food [23].

In view of the above, the present study was aimed at filling a literature gap about adolescents’ acceptance of a new food rich in β-glucans, obtained from a sustainable ingredient, that could potentially have a positive health effect. The specific objectives were to: (a) Evaluate adolescents’ acceptability of a flat bread modified by substituting part of the wheat flour content with a *P. ostreatus* powder rich in β-glucans and a conventional flat bread; and (b) investigate whether food technology neophobia and adolescents’ food habits could affect hedonic perception of the developed product.

Flat bread was chosen as the model system for *P. ostreatus* powder addition since it has been reported that cereal-based foods (e.g., bread and biscuits) represent the main important source of energy in school children and adolescents [24]. Moreover, cereal-based products are often used in school feeding programs in low and middle income countries and, thus, fortification of such products could have important health impacts.

## 2. Materials and Methods

### 2.1. Participants

A total of 285 adolescents, enrolled in 15 different high schools in northern Italy, were invited to participate in the experimental sessions during the XVI event of BergamoScienza, a science festival held every year in Bergamo (Italy). The purpose of this festival is to promote and disseminate the importance of science to civil society.

Two hundred and two adolescents (age range: 13–18 years, average age: 16.1 years, SD: 1.4; girls: 49.5%; boys: 50.5%) completed all the evaluations. The exclusion criteria were: Subjects who did not like flat breads (3.5%), subjects suffering from food intolerances and allergies (8%), subjects who were on medical treatment that could modify their taste perception (1.5%), and adolescents that did not complete all the evaluations (13%). Parents were informed about the procedures and were asked to sign an informed consent form when they agreed on participation. Only the adolescents with a signed informed consent form were included in the study. The consensus rate was 89% and questionnaires return rate was 71%. The study was conducted according to the guidelines established by the ethical committee in the Declaration of Helsinki.

### 2.2. Samples

#### 2.2.1. Mushroom Samples

A dried sample of *P. ostreatus* was produced by Società Agricola IoBoscoVivo srl (Vergiate, Varese, Italy), which holds a mycotheca of various basidiomycetes strains. *Agarigus bisporus*, which is a widespread mushroom, was chosen to compare the total glucans and β-glucans content between the different basidiomycetes considered. Two different samples of *A. bisporus* were purchased in the dried form on the market.

#### 2.2.2. Food Samples

Flat bread was chosen as the target food for *P. ostreatus* addition due to its common consumption as a snack or meal substitute among young people.

The ingredients of the novel sample, with added *P. ostreatus* (P), were as follows: 192 g of commercial soft wheat flour, 8 g of *P. ostreatus* powder, 25 g of extra virgin olive oil, 2 g of salt, 0.5 g Na_2_CO_3_ and 90 g of water. The control sample (C) was prepared by replacing the *P. ostreatus* powder with soft wheat flour. In both cases, the resulting dough was baked at 200 °C for 30 minutes.

### 2.3. Determination of β-Glucans Content

The total glucans content and β-glucans content were determined in triplicate using the Megazyme assay kit (K-YBGL, Megazyme International Ireland Ltd., Wicklow, Ireland), following the H_2_SO_4_ acid hydrolysis procedure by McCleary and Draga [10]. Insoluble β-glucan-rich fractions (pleuran) were obtained as described by Karacsonyi and Kuniak [25].

### 2.4. Food Technology Neophobia Scale (FTNS) Assessment

The FTNS questionnaire [19] translated into Italian according to Cattaneo and collaborators [26] was used since this tool has been reported to be a valid instrument to investigate subjects’ attitudes toward new food technologies [19]. The questionnaire consisted of 13 items, wherein each statement offered seven graded alternative responses, from “strongly disagree” (1) to “strongly agree” (7). Four of the 13 items reflect food neophilia; thus, these responses have to be reversed in order to calculate the final neophobia score. The FTNS score was calculated as the sum of the participant’s answers to each statement, yielding a range from 13 to 91. Higher scores indicate a higher food technology neophobia level.

### 2.5. Adolescent Food Habits Checklist (AFHC) Assessment

In order to investigate adolescents’ food habits, they were asked to complete the AFHC [22], a tool developed for this specific target population with items generated by dietitians and health psychologists, and selected by a pilot group of adolescents. This questionnaire has been reported as an efficient instrument to evaluate the healthy eating behavior in young consumers. The 23 items of the checklist related to fruit, vegetables and energy dense foods consumption, and were translated into Italian (see Supplementary Materials). Each item could be scored as true/false, or not applicable. The final score is calculated by assigning a one-point value for each healthy answer (e.g., “I usually avoid eating fried foods”; true = 1, false = 0). The final score is adjusted for not applicable and missing answers using the formula: Number of healthy response choices x (23/numbers of items completed). Higher scores indicate healthier eating behavior.

### 2.6. Overall Liking Evaluation

Participants were asked to taste the products monadically and to express their liking using a 10 cm visual analogue scale (VAS) anchored by the extremes “extremely disliked” (rated 0) and “extremely liked” (rated 10). Instructions for the use of the scale were given to the participant prior to the tasting [27].

### 2.7. Experimental Procedure

The evaluation was performed in a dedicated place during the XVI event of BergamoScienza in the presence of a teacher. All subjects were invited to take part in one session that took approximately 30 min. Firstly, the participants were asked to fill in the FTNS and AFHC questionnaires. The participants were then informed about the ingredients of the two flat bread samples that they were going to taste and, after tasting, they were asked to evaluate overall liking. The samples were presented randomly in plastic dishes coded with 3-digits numbers. All samples were prepared on the same day of the session and approximately 30 g of each sample was provided to the participants at room temperature. Participants were instructed not to share food with each other. The teachers monitored the participants to ensure that they did not influence each other.

### 2.8. Statistical Analysis

The total glucans and β-glucans contents of the mushroom samples were analyzed using one-way ANOVA with the least significant difference (LSD) as a multiple range test.

The internal reliability of the AFHC and FTNS was evaluated using Cronbach’s alpha. Responses to the FTNS questionnaire were subjected to, according to other previous approaches [26,28], principal components analysis with promax rotation and calculation of Cronbach’s alpha. Factorability of the sample was tested by the Kaiser–Meyer–Olkin Index and by Barlett’s sphericity test.

Firstly, ANOVAs were performed on FTNS and AFHC scores considering ‘age’ (≤16-year-old; >16-year-old), ‘gender’ (boys; girls) and their two-way interactions as factors.

Subsequently, a multifactor ANOVA was carried out on overall liking data considering ‘samples’ (sample P: added with *P. ostreatus* powder; sample C: control), ‘age’, ‘gender’ and their two-way interactions as factors. A similar model was applied to investigate the influence of FTNS level, as well as the AFHC level on liking scores. Correlation between FTNS and AFHC scores was also examined using Pearson’s correlation coefficient. A p-value of <0.05 was considered significant. All the analyses were performed using IBM SPSS Statistics for Windows, Version 24.0 (IBM Corp., Armonk, NY, USA).

## 3. Results

### 3.1. β-Glucans Content and Product Design

As shown in Table 1, β-glucans represent the major component of total glucans for both basidiomycetes evaluated, *P. ostreatus* and *A. bisporus*. Both the total content of glucans (37.4 g/100 g d.w) and content of β-glucans (35.6 g/100 g d.w.) in *P. ostreatus* were significantly higher than the total glucans (range 6.7–7.8 g/100 g d.w) and β-glucans (range 4.72–6.6) in *A. bisporus*. The level of β-glucans found in *P. ostreatus* may be considered high with respect to the range found in previous research [14]. The content of insoluble β-glucans (pleuran) in *P. ostreatus* was found to be 12 g/100 g d.w.

Hence, a 100 g portion of flat bread added with *P. ostreatus* could provide 1.0 g of β-glucans and 330 mg of pleuran, which can potentially provide health benefits considering the dosage used in in vivo studies [13,14,15].

### 3.2. Food Technology Neophobia Assessment

The evaluation of Cronbach’s α for the 13 items in the FTNS assessment showed a low level of internal consistency (Cronbach’s α = 0.64), as well as a low total explained variance from the principal components analysis (48%). Item 13 (“The media usually provides a balanced and unbiased view of new food technologies”) showed the lowest loading (<0.40); thus, it was removed from the analysis in order to increase the internal reliability of the scale. The removal of this item brought an increase in internal consistency (Cronbach’s α = 0.70) and a higher explained variance (52%).

The final FTNS score was recalculated as the sum of ratings given to the 12 statements, yielding a range from 12 to 84. Results of the Kaiser–Meyer–Olkin test (KMO = 0.77) and Barlett’s sphericity test (χ^2^ = 967.56; *p* < 0,001) showed adequacy of the sample for factor analysis.

The principal components analysis with promax rotation resulted in three distinct conceptual sets. The first component explained 27.9% of the total variation, being composed of items 1, 3, 4, and 5, defined by Cox and Evans [23] as “new food technologies are unnecessary”, as well as items 6 and 9, associated with the “perceptions of risk”, and 7 and 8, defined as “healthy choice”. The second component explained a further 14.5% of the variation and was positively associated with the reversed items 11 and 12. These two items (11 and 12), described separately from the others, reflected a neophilic attitude. The third component explained a further 9.6% of the variance and was associated with item 2. The general attitude towards technology and how its benefits and risks are perceived was investigated by means of the 12 items of the FTNS (Table 2).

The Food Technology Neophobia Scale (FTNS) mean value was 48.5 ± 0.6. No significant differences in the FTNS score among adolescents according to gender (F_(1,198)_ = 0.03, *p* = 0.89), age (F_(1,198)_ = 0.52, *p* = 0.47) and their interaction (F_(1,198)_ = 0.13, *p* = 0.91) were found.

### 3.3. Adolescent Food Habits Checklist Assessment

The internal reliability (Cronbach’s α) of the AFHC was 0.73. The average score calculated resulted in 11.9 ± 0.3 (range 1 to 23) showing generally slightly unhealthy behavior. A significant difference was found according to gender (F_(1,198)_ = 4.11; *p* < 0.05). In particular, female adolescents showed significantly healthier eating behavior (M = 12.7 ± 0.4) than males (M = 11.3 ± 0.3). No significant differences were found for the main factor, age (F_(1,198)_ = 0.98, *p* = 0.32), or the interaction between age and gender (F_(1,198)_ = 0.02; *p* = 0.88).

### 3.4. Overall Liking Evaluation

ANOVA results showed a significant effect of the main factor ‘samples’ (F_(1,397)_ = 14.18; *p* < 0.001) on liking scores. The sample with added *P. ostreatus* powder (sample P) was significantly preferred (M = 6.6 ± 0.2) by adolescents than the sample without mushroom powder (sample C, M = 5.7 ± 0.1).

**Figure 1:**
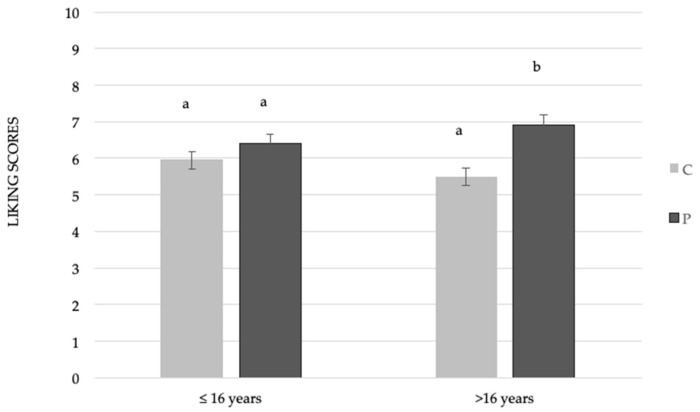
Mean liking scores ± SEM by samples according to age. Different letters indicate significant differences according to a post-hoc test for each sample.

A significant effect of the interaction between sample and age was found (F_(1,397)_ = 3.82; *p* < 0.05). As reported in Figure 1, adolescents >16 years gave significantly lower liking scores to the control sample (M = 5.5 ± 0.26) than the sample with mushroom powder added (M = 6.9 ± 0.2), whereas adolescents <16 years gave comparable liking scores to both samples (M_C_ = 5.9 ± 0.1; M_P_ = 6.4 ± 0.2).

No significant gender or gender × sample effects were found on liking scores (F_(1,397)_ = 2.45, *p* = 0.12; F_(1,397)_ = 0.04, *p* = 0.84; respectively).

### 3.5. Effect of Food Technology Neophobia and Adolescent Food Habits on Liking Scores

Subjects were divided into three groups according to their food technology neophobia level. The group with low food technology neophobia (23.8%) corresponded to the Neophilics and had a FTNS score within the lowest quartile (FNTS score ≤ 44). The medium FTNS group (Neutrals) accounted for 50% of the total sample and included subjects between the second and third quartiles (44 < NTFS score < 54). The group with high food technology neophobia (Neophobics) corresponded to 26.2% of the total sample and had a score within the highest quartile (FTNS ≥ 54). This classification method has been previously used in the evaluation of food neophobia in children [29] and adults [30].

A significant effect of food technology neophobia level on the liking score of samples was found (F_(2,389)_ = 13.34, *p* < 0.001).

**Figure 2:**
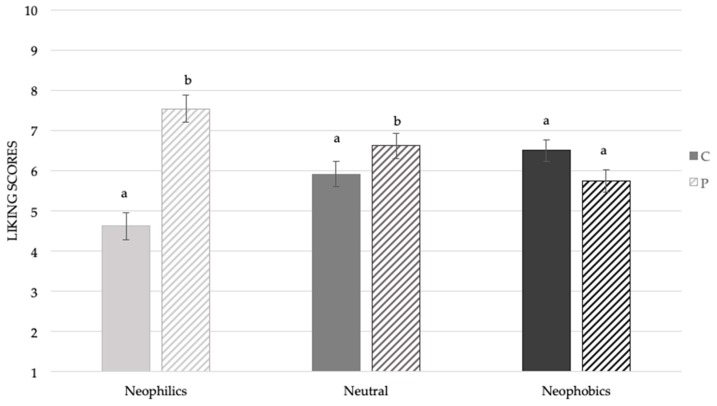
Mean liking scores ± SEM by samples according to food technology neophobia levels. Different letters indicate significant differences according to a post-hoc test for each sample.

Considering Figure 2, it is possible to observe that the Neophilic subjects gave significantly higher liking scores to the sample added with mushroom powder (M = 7.4 ± 0.4) compared to the control (M = 4.6 ± 0.3). A similar trend was highlighted in the Neutral subjects, even if the differences in hedonic perception were less pronounced compared to the Neophilic group of subjects (M_P_ = 6.7 ± 0.2; M_C_ = 5.9 ± 0.3). Neophobic subjects showed no difference in the acceptability of the samples.

Subjects were also classified according to their food habits in: Unhealthy behavior (27.7%, subjects with AFHC score within the lowest quartile ≤8.36), Neutral behavior (42.1%, AFHC score between the second and third quartiles 8.36 < AFHC < 15.8) and Healthy behavior (30.2%, subjects with AFHC score within the highest quartile ≥15.8).

**Figure 3:**
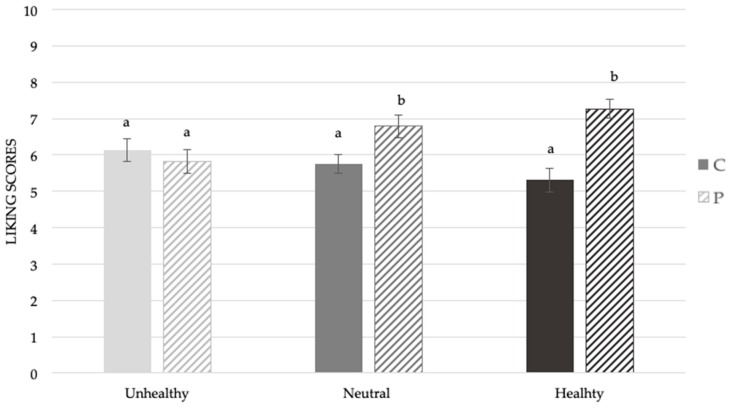
Mean liking scores ± SEM by samples according to food habits. Different letters indicate significant differences according to a post-hoc test for each sample.

A significant effect of food habits on the liking score of samples was found (F_(2,389)_ = 13.34, *p* < 0.001). In Figure 3**,** it is possible to observe that subjects characterized by Unhealthy eating behavior gave comparable hedonic scores to the two samples (M_P_ = 5.8 ± 0.3; M_C_ = 5.6 ± 0.2). Contrarily, subjects showing a Healthy eating behavior gave significantly higher liking scores to the sample with mushroom powder added (M = 7.3 ± 0.3) compared to the control (M = 5.4 ± 0.2). A similar trend was also highlighted in the Neutral subjects, even if the differences in hedonic perception were less pronounced compared to the Healthy eating group (M_P_ =6.8 ± 0.2; M_C_ = 6.1 ± 0.3).

A significant negative correlation (*r* = −0.20, *p* < 0.01) was also highlighted between FTNS and AFHC scores. Thus, subjects showing low food technology neophobia appeared to have healthier behavior compared to highly neophobic subjects.

## 4. Discussion

In the present study, the acceptance of a novel flat bread, rich in β-glucans due to the addition of *P. ostreatus* powder, was compared against a traditional flat bread in a specific target population of adolescents. The adolescents’ neophobia toward new food technologies and their healthy behavior was also investigated to understand whether these variables could influence the acceptance of the developed samples.

Most of the studies related to the evaluation of the sensory proprieties of food with *P. ostreatus* added concern adult consumers, whereas this study is the first of its kind to consider adolescents. We targeted this group of consumers due to low fiber consumption reported in young people, which is a purported factor in the development of the overweight condition [3]. Thus, developing new cereal-based food formulations well accepted from a sensory point of view is important in order to improve the daily diet quality of adolescents. The substitution of a part of wheat flour in the model food was performed in order to improve the nutritional profile of the flat bread. Indeed, besides the high β-glucans content, *P. ostreatus* powder is rich in several other nutrients and micronutrients (e.g., high quality protein and vitamin D), as well as being poor in fats and salt [17].

The present study demonstrated that it is possible to substitute part of the wheat flour of a baked product with *P. ostreatus* powder without negatively affecting its acceptability by young consumers—even increasing it. This result was not necessarily expected since consumers’ responses to new food that may be perceived as unfamiliar are often not positive [31]. The higher acceptance of the β-glucans-enriched sample may be explained by the sensory properties of *P. ostreatus* that have been reported to be characterized by an intense umami taste [32] and several volatile compounds, such as 1-octen-3ol and 3-octanol, responsible for the peculiar mushroom odor and flavor [17].

Controversial results are reported in literature about the effect of *Pleurotus* spp addition in different food matrices on sensory acceptability. Most of the reported data indicate a decrease in hedonic responses associated with the addition of mushroom powder in different food formulations (e.g., pasta, biscuits and vegetable soups). For example, in a previous study on vegetable soups enriched with *P. ostreatus* powder, we showed a decrease in liking scores with an increase in the concentration of mushroom [33]. Similarly, other studies have revealed that biscuits with high amounts of mushroom powder (12%) incorporated obtained the lowest score for overall acceptability compared to samples without the addition of powder [34]. However, low concentrations of mushroom powder usually do not adversely affect sensory acceptability. For example, vegetable soup enriched with 2% *P. ostreatus* and fortified bread with a low concentration of powder (up to 5%) have been reported to be suitable to develop well accepted products, showing the importance of the amount of powder used in the formulation [33,35]. Accordingly, the present results demonstrated that the fortification level at 2% addition of *P. ostreatus* positively affected young consumers’ hedonic responses. Considering the barriers to the acceptance of functional products by consumers, the results of the present study are interesting due to the higher acceptance of a functional food fortified in β-glucans, rather than a traditional one. It is important to take into account the specific target population involved in this study. Indeed, even if adolescents’ dietary intake is likely to reflect foods available at home and at school, as well as the influence of parents, they are already able to make their own food choices and, due to economic resources, they often buy snacks [22]. Since food habits directly affect nutritional status, it is of crucial importance to guide adolescents towards healthier food choices [36,37] in order to prevent overweight and obesity, which are constantly increasing over time and tend to persist in adulthood. In this context, a higher consumption of fiber could be an important factor to prevent the development of these pathologies.

However, developing new formulations is challenging since consumers are not always ready to compromise their sensory satisfaction with healthy benefits. Indeed, it has been demonstrated that consumers often relate healthiness with tastelessness [38], leading to a low acceptance of healthy foods. In this context, the proposed β-glucans-enriched flat bread appeared to satisfy both the nutritional and sensory aspects.

It should be considered that even if the sample added with mushroom powder was generally well accepted, there were different hedonic responses among adolescents according to their food technology neophobia level, which—for the first time—was investigated in a group of young consumers. Adolescents who mistrust new food technologies appeared to perceive the enriched products comparable to the control, while neutral and neophilic subjects significantly appreciated the sample with mushroom powder more than the traditional one. These results are in line with the general reported reluctance to try new and unfamiliar food of subjects characterized by a high food neophobia level [39]. In this context, previous findings have highlighted a negative correlation between willingness to try various unfamiliar foods (including a functional food, for example margarine with plant sterols) and food neophobia in Australian adolescents [40]. Interestingly, the present results demonstrated that flat bread with *P. ostreatus* added was satisfactorily accepted, even by neophobic subjects. Indeed, it has been reported that cereal-based products, such as pasta and bread, are usually more liked and consumed than fruit and vegetables, even by subjects showing a high food neophobia level [30,41].

In line with previous research [22], the adolescents tested in the present study showed a low level of healthy eating behavior. Moreover, adolescents showing a healthier eating behavior gave significantly higher hedonic scores to the sample enriched with β-glucans compared to the subjects showing an unhealthy eating behavior. These outcomes underline the importance of educating young people on healthy lifestyles, which, in turn, makes them more likely to appreciate functional foods with potential health benefits.

One limitation of the study should be mentioned. It would have been interesting to have included a sensory description of the samples for the purpose of having a better understanding of the sensory attributes directly related to the acceptance of the mushroom powder-enriched sample.

In conclusion, this study suggests that it is possible to develop a β-glucans-enriched product appreciated by adolescent consumers. The FNTS and AFHC tools were useful for categorizing the adolescents according to their mistrust in new technologies and their food habits, highlighting the effect of these behavioral features on the acceptability of food products. Indeed, the developed product appeared to be suitable for consumption by adolescents, especially those with a healthy eating behavior and trust in new food technologies. Thus, this fortified food may be one of the matrices used to achieve the daily recommended intake of β-glucans by adolescents over time, thereby counteracting the phenomenon of the overweight condition and obesity.

## Figures and Tables

**Table 1:** Content of total glucans (mean ± *SD*, g/100 g d.w.) and β-glucans (mean ± *SD* g/100 g d.w.) in *P. ostreatus and A. bisporus.*

Samples	Total Glucans	β-Glucans
*P. ostreatus*	37.4 ± 1.6 ^a^	35.6 ± 1.1 ^a^
*A. bisporus* (batch 1)	6.7 ± 0.2 ^b^	4.72 ± 0.3 ^b^
*A. bisporus* (batch 2)	7.8 ± 0.4 ^b^	6.6 ± 0.4 ^b^

^a,b^ Different letters in the same column represent significant differences (least significant difference, LSD; *p* < 0.05).

**Table 2:** Food Technology Neophobia Scale: Explained variance (%), factor loadings, items mean and standard deviation. R = reversed items.

Items	PC1 (27.9%)	PC2 (14.5%)	PC3 (9.6%)	Mean (SD)
1. There are plenty of tasty foods around so we don’t need to use new food technologies to produce more.	**0.75**	−0.18	0.06	3.9 (1.5)
2. The benefits of new food technologies are often grossly overstated.	−0.07	0.02	**0.78**	4.4 (1.2)
3. New food technologies decrease the natural quality of food.	**0.49**	0.35	0.20	4.3 (1.6)
4. There is no sense trying out high-tech food products because the ones I eat are already good enough.	**0.70**	−0.22	−0.10	3.8 (1.6)
5. New foods are not healthier than traditional foods.	**0.54**	0.20	0.26	4.2 (1.6)
6. New food technologies are something I am uncertain about.	**0.55**	−0.07	−0.09	4.1 1.4)
7. Society should not depend heavily on technologies to solve its food problems.	**0.74**	0.03	−0.04	4.4 (1.5)
8. New food technologies may have long term negative environmental effects.	**0.68**	0.15	−0.08	4.3 (1.5)
9. It can be risky to switch to new food technologies too quickly.	**0.61**	−0.13	−0.01	4.4 (1.4)
10. New food technologies are unlikely to have long term negative health effects. (R)	0.08	0.24	**−0.77**	4.0 (1.5)
11. New products produced using new food technologies can help people have a balanced diet. (R)	−0.08	**0.83**	−0.01	3.4 (1.4)
12. New food technologies give people more control over their food choices. (R)	−0.11	**0.76**	−0.17	3.5 (1.2)

Loadings greater than |0.4| are shown in bold.

## Supplementary Materials

The following are available online at https://www.mdpi.com/2304-8158/8/10/433/s1, Table S1: English and Italian version of the Adolescence Food Habits Checklist.
