# Supplementary material for: Acceptance of a New Food Enriched in β-Glucans among Adolescents: Effects of Food Technology Neophobia and Healthy Food Habits
Source: Foods. 2019 Sep 23;8(10):433. doi: 10.3390/foods8100433 (PMC6835998; doi:10.3390/foods8100433)
Supplement: Supplementary file 1 [file foods-08-00433-s001.docx]

**Table S1.** English and Italian version of the Adolescence Food Habits Checklist.

| **Item** | **English version** | **Italian version** |
| --- | --- | --- |
| 1 | If I am having lunch away from home, I often choose a low-fat option.  *True or False or I never have lunch away from* *home* | Quando devo pranzare fuori casa scelgo alimenti a basso contenuto di grassi.  *Vero o Falso o Non pranzo mai fuori casa* |
| 2 | I usually avoid eating fried foods.  *True or False* | Di solito cerco di non mangiare cibi fritti.  *Vero o Falso* |
| 3 | I usually eat a dessert or pudding if there is one available.  *True or False* | Di solito mangio qualcosa di dolce se è disponibile in casa.  *Vero o Falso* |
| 4 | I make sure I eat at least one serving of fruit a day.  *True or False* | Cerco di mangiare almeno un frutto al giorno.  *Vero o Falso* |
| 5 | I try to keep my overall fat intake down.  *True or False* | Cerco di non mangiare cibi troppo ricchi in grassi. *Vero o Falso* |
| 6 | If I am buying crisps, I often choose a low-fat brand.  *True or False or I never buy crisps* | Se compro delle patatine, cerco di comprarle a basso contenuto di grassi.  *Vero o Falso o Non compro mai patatine* |
| 7 | I avoid eating lots of sausages and burgers.  *True or False or I never eat sausages or burgers* | Cerco di non mangiare troppi hamburger o wurstel.  *Vero o Falso o Non mangio mai hamburger o wurstel* |
| 8 | I often buy pastries or cakes.  *True or False* | Spesso compro torte o merendine.  *Vero o Falso* |
| 9 | I try to keep my overall sugar intake down.  *True or False* | Cerco di non mangiare troppi alimenti ricchi in zuccheri.  *Vero o Falso* |
| 10 | I make sure I eat at least one serving of vegetables or salad a day.  *True or False* | Faccio in modo di mangiare almeno una porzione di verdura al giorno.  *Vero o Falso* |
| 11 | If I am having a dessert at home, I try to have something low in fat.  True=False=I don’t eat desserts | Se a casa mangio dei dolci cerco di sceglierli a basso contenuto di grassi.  *Vero o Falso* *o Non mangio dolci* |
| 12 | I rarely eat takeaway meals.  *True or False* | Compro raramente cibi da asporto.  *Vero o Falso* |
| 13 | I try to ensure I eat plenty of fruit and vegetables.  *True or False* | Cerco di mangiare molta frutta e verdura  *Vero o Falso* |
| 14 | I often eat sweet snacks between meals.  *True or False* | Spesso mangio merendine tra un pasto principale e l’altro. *Vero o Falso* |
| 15 | I usually eat at least one serving of vegetables (excluding potatoes) or salad with my evening meal. *True or False* | Di solito mangio una porzione di verdura (escluse le patate) durante la cena.  *Vero o Falso* |
| 16 | When I am buying a soft drink, I usually choose a diet drink.  *True or False* *or I never buy soft drinks* | Quando compro bibite gassate scelgo bibite dietetiche.  *Vero o Falso* Non compro mai bibite gassate |
| 17 | When I put butter or margarine on bread, I usually  spread it thinly.  *True or False* *or I never have butter or margarine on bread* | Quando metto il burro o la margarina sul pane lo spalmo in uno strato sottile.  *Vero o Falso* o *Non metto mai burro o margarina sul pane* |
| 18 | If I have a packed lunch, I usually include some chocolate and/or biscuits.  *True or False* *or I never have a packed lunch* | Se mangiassi il pranzo al sacco includerei del cioccolato o dei biscotti.  *Vero o Falso* *o Non mangio mai pranzo al sacco* |
| 19 | When I have a snack between meals, I often choose  fruit.  *True or False* *or I never eat snacks between meals* | Quando faccio merenda tra i pasti principali spesso scelgo di mangiare la frutta.  *Vero o Falso* *o Non faccio mai merenda tra i pasti principali* |
| 20 | If I am having a dessert or pudding in a restaurant, I  usually choose the healthiest one.  *True or False* *or I never have desserts in restaurants* | Se mangio un dessert/dolce quando sono al ristorante cerco di scegliere il più salutare.  *Vero o Falso o Non mangio mai il dolce quando sono al ristorante* |
| 21 | I often have cream on desserts.  *True or False* *or I don’t eat desserts* | Spesso quando mangio i dolci aggiungo della panna.  *Vero o Falso* *o Non mangio mai dolci* |
| 22 | I eat at least three servings of fruit most days.  *True or False* | Cerco di mangiare tre porzioni di frutta al giorno.  *Vero o Falso* |
| 23 | I generally try to have a healthy diet.  *True or False* | Cerco di avere una dieta il più salutare possibile.  *Vero o Falso* |
